# Training load and pain response during progressive resistance training in patients with hip osteoarthritis in the PROHIP trial

**DOI:** 10.1016/j.ocarto.2025.100690

**Published:** 2025-10-04

**Authors:** Emma Smed Bryld, Laura Christiansen, Kim Gordon Ingwersen, Søren Overgaard, Lone Ramer Mikkelsen, Inger Mechlenburg, Thomas Frydendal

**Affiliations:** a Department of Orthopedic Surgery, Aarhus University Hospital, Aarhus, Denmark; b Department of Clinical Medicine, Aarhus University, Aarhus, Denmark; c Department of Physiotherapy, Lillebaelt Hospital – University Hospital of Southern Denmark, Vejle Hospital, Vejle, Denmark; d Department of Regional Health Research, University of Southern Denmark, Odense, Denmark; e Department of Orthopedic Surgery and Traumatology, Copenhagen University Hospital Bispebjerg, Copenhagen, Denmark; f Department of Clinical Medicine, Faculty of Health and Medical Sciences University of Copenhagen, Copenhagen, Denmark; g University Clinic for Orthopedic Pathways (UCOP), Elective Surgery Centre, Regional Hospital Silkeborg, Silkeborg, Denmark; h Exercise Biology, Department of Public Health, Aarhus University, Aarhus, Denmark; i Research Center for Activity and Prevention, VIA University College, Aarhus, Denmark; j Department of Clinical Research, University of Southern Denmark, Odense, Denmark

**Keywords:** Hip osteoarthritis, Exercise, Resistance training, Numerical rating scale, Hip pain

## Abstract

**Objective:**

To describe training load and repetitions, illustrate the pre- and post-exercise hip pain intensity trajectories throughout a 12-week progressive resistance training (PRT) program, and evaluate the difference in change in pre-exercise hip pain intensity between high and moderate-to-low adherence to PRT among individuals with severe hip osteoarthritis and indication for surgery.

**Design:**

Secondary analysis of a randomized controlled trial reporting data only from participants who were enrolled from September 2019 through June 2021 from four orthopedic departments in Denmark and assigned to PRT. Patient-reported hip pain intensity at rest was measured before and after each session using a Numerical Rating Scale (NRS) ranging from 0 (no pain) to 10 (worst pain imaginable). High adherence was defined as attending at least 18 of 24 scheduled sessions.

**Results:**

A total of 55 participants (mean age 67.7 years and 51 ​% females) received PRT. Training load increased while repetitions decreased from week 1 to week 12. The mean pre-exercise NRS decreased from 3.18 points at baseline to 2.13 points at 12-weeks (difference, 1.05 points [95%CI 0.46, 1.65]). The mean post-exercise NRS decreased from 2.87 points at baseline to 2.04 points at 12-weeks, (difference, 0.83 points [95%CI 0.30, 1.37]). There was no significant difference in mean change in pre-exercise NRS scores between high and moderate-to-low adherence (group difference, 1.08 points [95%CI -0.85, 3.02]).

**Conclusions:**

Individuals with severe hip osteoarthritis and indication for surgery can exercise at progressively higher intensity while maintaining low hip pain intensity. High adherence did not result in greater pain relief.

**Clinical trial registration number:**

NCT04070027.

## 1 Introduction

Hip osteoarthritis is a major contributor to disability globally [1]. We recently reported results from the Progressive Resistance Training versus Total Hip Arthroplasty (PROHIP) randomized trial, which demonstrated that total hip arthroplasty led to clinically meaningful improvements in patient-reported hip pain and function at 6 months as compared with progressive resistance training (PRT) [2]. Still, exercise is recommended as part of first-line treatment for hip osteoarthritis regardless of pain, age and/or severity of disease [3], and PRT appears to provide moderate reductions in hip pain and improvements in hip function even in individuals with indication for total hip arthroplasty [4,5]. Even for those patients undergoing total hip arthroplasty, supervised PRT seem beneficial for faster postoperative recovery [6,7].

However, detailed insights on how training load, repetitions, and pain intensity evolve during a supervised PRT program is lacking. Understanding the trajectory of hip pain intensity during an exercise program is crucial for both patients and clinicians. Since this knowledge may influence and improve treatment adherence and outcomes.

This study aimed to provide a detailed description of exercise load and repetitions, illustrate the pre- and post-exercise hip pain intensity trajectories throughout a 12-week PRT program, and evaluate the difference in change in pre-exercise hip pain intensity at rest between high and moderate-to-low adherence among individuals with severe hip osteoarthritis and indication for surgery.

## 2 Methods

### 2.1 Study design

This study was designed as a cohort study, as it is a secondary analysis of a randomized controlled trial, using data on hip pain intensity, training load and repetitions trajectories only from the participants randomly assigned to the resistance training group in the PROHIP trial [2].

The PROHIP trial was approved by the Regional Committees on Health Research Ethics for Southern Denmark (Project-ID: S-20180158) and the Danish Data Protection Agency (Journal No 19/20337), registered at ClinicalTrials.gov (NCT04070027). The present study was reported in agreement with the Strengthening the Reporting of Observational Studies in Epidemiology (STROBE) statement for longitudinal studies [8].

### 2.2 Setting and participants

Participants were recruited from September 2019 through June 2021 from the orthopedic departments at Vejle Hospital, Odense University Hospital, Aarhus University Hospital and Næstved Hospital in Denmark. Individuals aged 50 years or older with severe hip osteoarthritis and indication for total hip arthroplasty based on hip pain, clinical presentation (i.e., symptom duration of at least 3 months, functional impairments, decreased range-of-motion, and attempted previous treatment with analgesics), and radiographic imaging (i.e., joint space width below 2 ​mm), as assessed by an orthopedic surgeon were eligible for enrolment. Exclusion criteria were severe walking deficits (dependency on two crutches or walker); body mass index (BMI) above 35 ​kg/m^2^; lower extremity fractures within the previous 12 months; planned other lower extremity surgery within 6 months; cancer and current chemo-, immune- or radiotherapy; neurological diseases, other reasons (e.g., inability to understand Danish, or being considered mentally or physically unable to participate). Research coordinators provided each participant with detailed standardized information about the PROHIP trial and obtained written informed consent [2,9,10].

### 2.3 Progressive resistance training

All participants were offered 12-weeks of PRT, with two 1-h sessions per week. Each training session were performed with individual supervision by a physiotherapist and at least 48 ​h of rest between sessions. The PRT program included a 10-min warm-up on a stationary bicycle followed by four exercises for the lower extremities (leg press, hip extension, hip flexion and hip abduction) performed unilaterally in machines or cable pulleys in three sets separated by 60 ​s of rest, with as full a range-of-motion as possible. Progression of training load followed a linear model of periodization, with an initial relative load of 12 repetition maximum (RM) for weeks 1–2, 10 RM in weeks 3–6 and 8 RM in weeks 7–12. The absolute training load was adjusted between each set by completing as many repetitions as possible until contraction to concentric muscle failure. Training load was increased if participants were able to perform two or more repetitions more than planned or decreased if less than eight repetitions were completed. The supervising physiotherapist registered attendance, training load in kilograms and number of repetitions for each set for all four exercises. High adherence was defined as attending at least 18 of 24 (≥75 ​%) PRT sessions. Detailed descriptions of the PRT program are available in the trial protocol [10].

### 2.4 Numerical rating scale and pain management

Patient-reported hip pain intensity at rest was assessed using an 11-point Numerical Rating Scale (NRS) ranging from 0 (no pain) to 10 (worst pain imaginable) [11]. The minimal clinically important change is estimated to be 1 point in individuals with hip osteoarthritis [12]. Participants rated their hip pain intensity for the affected hip before and after each exercise session from baseline (session 1) to week 12 (session 24) in a seated position at rest.

We used a previously described pain management model [12], in which hip pain was accepted during the exercises and was used to monitor and guide progression and regression of absolute training load between sessions during the 12-weeks of PRT. Hip pain intensity ratings from 0 to 2 was considered as ‘safe’, 3 to 5 as ‘acceptable’, and above 5 as ‘high risk’. The following day after an exercise session, participants hip pain should subside to pain ‘as usual’ otherwise the training load was reduced in the following exercise session [13].

### 2.5 Statistics

We assessed the assumption of normality of the continuous variables using visual inspection of histograms and quantile-quantile plots. We used a paired student's t-test to compare the mean difference in pre- and post-exercise hip pain intensity from baseline to 12-weeks follow-up.

To evaluate whether adherence to PRT influenced hip pain intensity, we used an unpaired Student's t-test to compare the change in mean pre-exercise hip pain intensity from baseline to 12-weeks follow-up between the high and moderate-to-low adherence groups.

Trajectories in training load and repetitions for all four exercises, as well as pre- and post-exercise NRS scores are presented. Changes from baseline and between-group differences (i.e., high versus low-to-moderate adherence) are reported with means and 95 ​% confidence intervals (95%CI). All statistical analyses were performed using R version 4.3.1 (the R Foundation for Statistical Computing, Vienna, Austria).

## 3 Results

In the PROHIP trial, 56 participants were enrolled and randomly assigned to the PRT group, with 55 participants receiving the intervention. The mean age of participants who received the intervention was 67.7 (SD 7.1) years, 51 ​% were females and mean BMI was 28.3 (SD 3.8) (Supplementary Table S1).

**Fig. 1:**
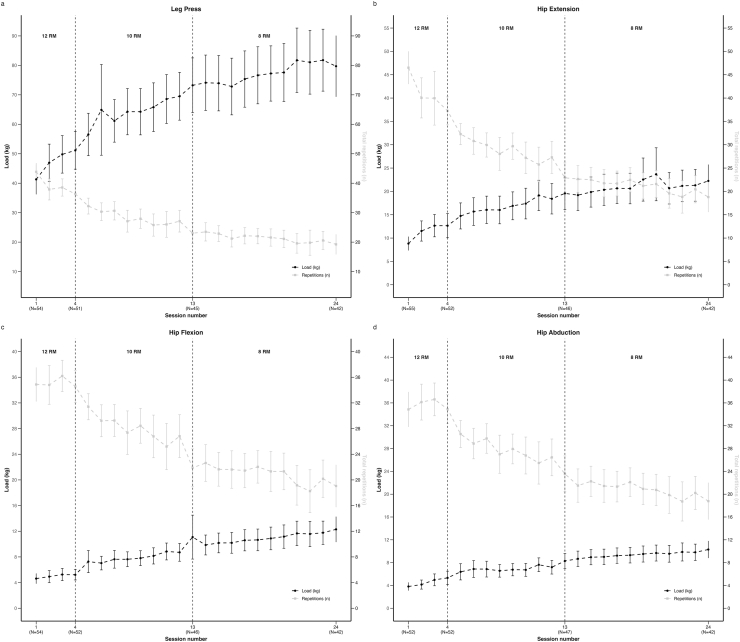
Trajectories of exercise load and total number of repetitions completed in all three sets for (A) leg press, (B) hip extension (C) hip flexion and (D) hip abduction throughout the 24 supervised sessions of the progressive resistance training program. The number of missing participants is due to dropouts, sessions not attended or exercises not performed due to pain. Values are means and error bars indicate 95 ​% confidence intervals (95 ​% CI).

The training load increased, and repetitions decreased from week 1 to week 12 in line with the prescribed linear progression model across all four exercises (Fig. 1A–D).

**Fig. 2:**
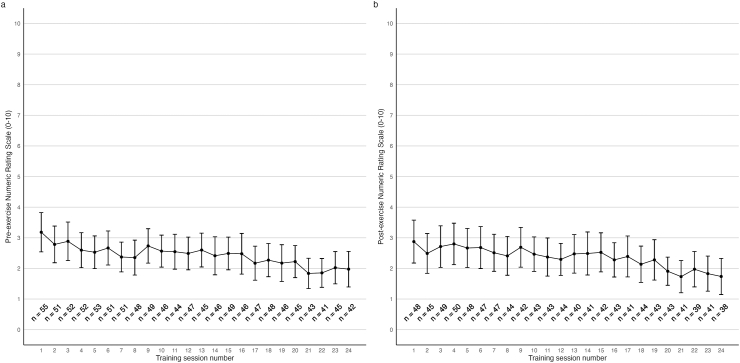
Trajectories in hip pain intensity at rest from each training session measured with the Numerical Rating Scale (NRS) ranging from 0 (no pain) to 10 (worst pain imaginable). (A) Pre-exercise hip pain intensity at rest. (B) Post-exercise hip pain intensity at rest. The number of missing participants is due to dropouts, sessions not attended or assessment not recorded. Values are means and error bars indicate 95 ​% confidence intervals (95 ​% CI).

The mean pre-exercise NRS declined from 3.18 points (95%CI 2.54, 3.82) at baseline to 2.13 points (95%CI 1.59, 2.66) at 12-weeks, corresponding to a reduction of 1.05 points (95%CI 0.46, 1.65). (Fig. 2A).

The mean post-exercise NRS declined from 2.87 points (95%CI 2.19, 3.54) at baseline to 2.04 NRS (95%CI 1.49, 2.58) at 12-weeks, corresponding to a reduction of 0.83 points (95%CI 0.30, 1.37). (Fig. 2B).

Among the 55 participants receiving PRT, 47 had high adherence and 8 had moderate-to-low adherence. The pre-exercise NRS declined by 1.21 points (95%CI 0.59, 1.83) in the high adherence group and by 0.13 points (95%CI -2.03, 2.28) in the moderate-to-low adherence group from baseline to 12-weeks, corresponding to a mean between-group difference in change of 1.08 points (95%CI -0.85, 3.02). For the majority of characteristics, there were observed negligible differences between the two adherence groups at baseline, but in the low-to-moderate adherence group there were a higher prevalence of females, fewer had an educational level above high school and an alcohol intake >10 units per week, and more had previously received supervised exercise, as well as reporting worse hip symptoms and hip-related quality of life than those in the high adherence group (Supplementary Table S1).

## 4 Discussion

During a 12-week PRT program for individuals with severe hip osteoarthritis and an indication for total hip arthroplasty, the training load increased, and repetitions decreased in line with the prescribed linear progression model across all four exercises, while mean hip pain intensity levels was maintained low within the safe and acceptable categories. Both hip pain intensity before and after exercise sessions decreased over the 12-weeks, and the observed reduction may potentially be considered clinically important. However, high adherence to the PRT program did not yield significantly greater reductions in pre-exercise hip pain intensity.

We are not aware of previous studies that have investigated hip pain intensity trajectories in individuals with severe hip osteoarthritis and indication for total hip arthroplasty during a PRT program. However, a previous study has described changes in pain intensity throughout a neuromuscular exercise program (NEMEX) in individuals with mild to moderate knee and hip osteoarthritis, and the study also reported a gradual decrease in hip pain intensity over the course of an exercise program [14]. While another study assessed this throughout a PRT program in individuals with severe knee osteoarthritis scheduled for total knee arthroplasty, which found no exacerbation of knee joint pain despite a substantial progression in training load and increased muscle strength [15]. These results from the previous studies are in line with our findings. This may indicate that similar reductions in hip pain intensity can be expected of different types of exercise and regardless of hip osteoarthritis severity.

Although our point estimate appear to suggest greater benefit for participants with high adherence to PRT as compared to those with moderate-to-low adherence, our 95%CI was wide possibly indicating no influence of adherence on hip pain intensity. This may be explained by low statistical power due to the small sample size of 8 participants in the low-to-moderate adherence group. On the other hand, in consistency with our findings, a previous study also reported no greater reduction in joint pain intensity among patients with mild to moderate knee and hip osteoarthritis who performed NEMEX with high adherence, as compared to those with low-to-moderate adherence [14].

This study has some limitations. The main limitation is the absence of a control group making it impossible to infer causality of PRT on hip pain intensity trajectories. Therefore, the observed reductions in hip pain intensity may be influenced by other factors such as natural disease progression (i.e., regression towards the mean), placebo response, or concomitant treatments. Another limitation is that there were indications of systematic differences between our high and moderate-to-low adherence groups with respect to some baseline characteristics, as these two groups were not randomly assigned. However, the majority of the characteristics did not differ between the two groups at baseline, suggesting this is probably unlikely to explain our findings. Although our descriptive analysis of pre- and post-exercise hip pain intensity trajectories included ratings from all 24 training sessions, not all participants attended every session and some assessments were missing. Some participants also stopped earlier due to an increase or no decrease in hip pain [2]. These factors may have introduced selection bias, potentially leading to an overestimation of the observed reductions in hip pain intensity before and immediately after exercise. On the other hand, since hip pain intensity ratings were available for between 38 and 55 participants per session through the 12-week program, with 46 participants having less than 20 ​% missing data across all sessions, this could indicate a minor influence on our estimates. Overall, due the observational design and current limitations our present findings should therefore be interpreted with caution.

In conclusion, individuals with severe hip osteoarthritis and indication for total hip arthroplasty can exercise at progressively higher intensity while maintaining low hip pain intensity levels. The 12-week PRT program was associated with a reduction in hip pain intensity both before and immediately after exercise that may be considered clinically important. High adherence to PRT did not result in greater relief in pre-exercise hip pain intensity. These findings provide detailed information about load progression and the hip pain trajectory during an exercise treatment. This information is helpful for clinicians as it can help educate and manage patients’ expectations when starting supervised resistance training for hip osteoarthritis.

## Author contributions

Conceived and designed the trial: KGI, SO, LRM, IM and TF. Collected and assembled data: TF, ESB and LC. Performed the statistical analysis ESB and LC. Drafted the first version of the manuscript: ESB, LC. Provided critical revision of the article for important intellectual content: KGI, SO, LRM, IM and TF. Approved the final manuscript: ESB, LC, KGI, SO, LRM, IM and TF. Obtained funding: KGI, SO, LRM, IM and TF.

## Role of the funding source

The Danish Rheumatism Association (R153-A4771, R161-A5280, R166-A5553, R176-A6231), Region of Southern Denmark (17/33622, 18/41994), Region Zealand (R23-A786), The Association of Danish Physiotherapists Research Fund (N/A), The Research Council at Næstved-Slagelse-Ringsted Hospitals (N/A), and The A.P. Moeller Foundation (N/A) have supported the trial. The funders were not involved in designing the study, data collection, data management, data analysis, interpreting the results, writing the article, or deciding to submit the article for publication.

## Declaration of competing interest

The authors declare that they have no competing interest.
