# Supplementary material for: Training load and pain response during progressive resistance training in patients with hip osteoarthritis in the PROHIP trial
Source: Osteoarthr Cartil Open. 2025 Oct 4;7(4):100690. doi: 10.1016/j.ocarto.2025.100690 (PMC12547905; doi:10.1016/j.ocarto.2025.100690)
Supplement: Multimedia component 1 [file mmc1.pdf]

SUPPLEMENTARY APPENDIX

Table S1. Baseline characteristics of the patients with severe hip osteoarthritis randomized to the progressive resistance training group receiving the intervention, and sub-groups based on adherence to the intervention. High adherence was defined as attending at least 18 of 24 (≥75%) sessions. *

Characteristic	High adherence (N = 47)	Moderate-to-low adherence (N = 8)	Progressive Resistance Training (N = 55)
Age – yr	67.8 ± 7.0	66.8 ± 8.2	67.7 ± 7.1
Female sex, no. (%)	23 (49)	5 (63)	28 (51)
Body-mass index	28.6 ± 3.8	26.8 ± 3.4	28.3 ± 3.8
Educational level above high school – no. (%)	26 (55)	2 (25)	28 (51)
Employment status – no. (%)			
Employed for wages or self-employed	13 (28)	2 (25)	15 (27)
On sick leave	1 (2)	1 (12)	2 (4)
Retired	33 (70)	5 (63)	38 (69)
Other	0	0	0 (0)
Current smoking – no. (%)	3 (6)	1 (12)	4 (7)
Alcohol intake of >10 units/wk – no. (%)†	6 (13)	0 (0)	6 (11)
Duration of hip symptoms – yr, median (IQR)	2.0 (0.8–4.0)	0.8 (0.5–1.7)	1.5 (0.7–4.0)
Previous total hip arthroplasty – no. (%)	8 (17)	1 (12)	9 (16)
Previous total knee arthroplasty – no. (%)	1 (2)	0	1 (2)
Previous treatment for hip-related pain – no. (%)			
Supervised exercise	13 (28)	3 (38)	16 (29)
Manual or passive treatment	8 (17)	1 (12.5)	9 (16)
Glucocorticoid injection	1 (2)	1 (12.5)	2 (4)
Other non-surgical treatment	7 (15)	1 (12.5)	8 (15)
Use of analgesic agents for hip-related pain – no. (%)			
Acetaminophen	36 (80)	8 (100)	44 (80)
Nonsteroidal anti-inflammatory drug	16 (34)	1 (12)	17 (31)
Opioid	3 (6)	0	3 (5)
Other analgesic agent	3 (6)	1 (12)	4 (7)
Oxford Hip Score ‡	25.5 ± 5.0	21.3 ± 7.7	24.8 ± 5.6
Hip Disability Osteoarthritis Outcome Score subscales §			
Pain	46.9 ± 16.7	41.9 ±15	46.2 ± 16.4
Symptoms	45.5 ± 16.2	36.3 ± 20.0	44.2 ± 16.9
Function in activities of daily living	53.3 ± 17.0	47.0 ± 22.4	52.4 ± 17.8
Hip-related quality of life	35.2 ± 14.8	25.0 ± 13.4	33.8 ± 15.0
Function in sports and recreation	31.8 ± 21.0	28.1 ± 18.6	31.3 ± 20.6
UCLA activity score ¶	5.3 ± 1.8	4.3 ± 0.9	5.1 ± 1.7
Gait speed in the 40-m fast-paced walk test – m/sec	1.5 ± 0.3	1.2 ± 0.4	1.4 ± 0.3
No. of repetitions in the 30-sec sit-to-stand test	10.5 ± 3.4	10.8 ± 3.7	10.6 ± 3.4

*Values are presented as number (%), median (interquartile range [IQR]), and mean (standard deviation). Percentages may not total 100 because of rounding.

† One unit equals 10 ml or 8 g of pure alcohol.

‡ The Oxford Hip Score is a measure of hip pain and function, providing a single total score from 0 to 48, with higher scores indicating less pain and better function.

§The Hip Disability Osteoarthritis Outcome Score comprises subscales for pain, symptoms, function in daily activities, hip-related quality of life, and function in sports and recreation, with each subscale ranging from 0 (worst) to 100 (best).

¶The University of California, Los Angeles (UCLA), activity score measures the level of physical activity on a scale from 1 (in active) to 10 (regular physical activity with high intensity).
